# “*The Right Way at the Right Time*”: Insights on the Uptake of Falls Prevention Strategies from People with Dementia and Their Caregivers

**DOI:** 10.3389/fpubh.2016.00244

**Published:** 2016-11-02

**Authors:** Claudia Meyer, Briony Dow, Keith D. Hill, Jean Tinney, Sophie Hill

**Affiliations:** ^1^Centre for Health Communication and Participation, School of Psychology and Public Health, La Trobe University, Bundoora, VIC, Australia; ^2^RDNS Institute, St Kilda, VIC, Australia; ^3^National Ageing Research Institute, Parkville, VIC, Australia; ^4^Centre for Health Policy, School of Global and Population Health, University of Melbourne, Parkville, VIC, Australia; ^5^School of Physiotherapy and Exercise Science, Curtin University, Perth, WA, Australia

**Keywords:** falls, dementia, caregivers, knowledge translation, qualitative

## Abstract

Strong evidence exists for effective falls prevention strategies for community-dwelling older people. Understanding the translation of these strategies into practice for people with dementia has had limited research focus. People with dementia desire to have their voice heard, to engage meaningfully in the health-care decision-making process, making it a priority for researchers and practitioners to better understand how to engage them in this process. This paper reports on the qualitative aspects of a series of studies, which aimed to identify the views of people with dementia and their caregivers regarding perceptions of falls prevention and the successes and challenges of adopting falls prevention strategies. Twenty five people with dementia and their caregivers were interviewed in their homes at baseline, and 24 caregivers and 16 people with dementia were interviewed at completion of a 6-month individualized falls prevention intervention. Interviews were audio-recorded, transcribed verbatim, and thematically analyzed. Five themes were identified at baseline: *perceptions of falls; caregivers navigating the new and the unpredictable; recognition of decline; health services – the need for an appropriate message; and negotiating respectful relationships*. At 6 months, caregivers and people with dementia decided on “*what we need to know*” with firm views that the information regarding falls risk reduction needed to be in “*the right way* … *at the right time*.” Rather than caregivers and people with dementia being only recipients of knowledge, they felt they were “*more than just empty vessels to be filled*” drawing on a “*variety of resources*” within their circle of influence to be able to positively “*adapt to change*.” The voices of people with dementia and their caregivers add an important dimension to understanding the translation of falls prevention knowledge for this population. Insights from this study will enable community care health professionals to understand that people with dementia and their caregivers can, and wish to, contribute to implementing falls prevention strategies through their resourcefulness and inclusion in the therapeutic partnership.

## Introduction

Dementia describes a set of symptoms related to cognitive decline, which affects over 300,000 Australians and is characterized by changes in memory, perception, and judgment (1). Falls are another well-recognized public health issue for community-dwelling older people, with approximately 30% of older people and 50–80% of people with dementia falling within a given year (2), people with dementia having a threefold risk of fracture (3). For the person with dementia, alterations in executive functioning may cause visuospatial changes; decrease in working memory; and changes in concentration/attention (4, 5), all of which potentially influence the ability of people with dementia to successfully adopt and implement falls prevention strategies.

There has been a recent focus on falls prevention for people with dementia, which includes recognition that simply replicating successful trials in this high falls risk population, shown to be effective in cognitively intact older people, may not be sufficient (6). There has also been a greater focus in recent falls prevention research on improving the uptake of, and adherence to, falls prevention strategies. Addressing the suboptimal uptake and lack of sustained participation in falls prevention interventions for older people is required to target reduction in the burgeoning injurious falls rates (7). Adherence to falls prevention interventions has been problematic (8), yet maximization of participation by older people can be enhanced through personally relevant and appropriate advice, and if they perceive a benefit (9–11), which is of equal importance to the person with dementia.

To maximize participation in falls prevention strategies, meaningful engagement needs to move beyond agreeing or adhering to advice and/or treatment to active and informed choice (12). Research with people with dementia has found that they desire to engage in their health care through having their voices heard (13). Guided by a person-centered philosophy for best practice dementia care, which values the unique needs and preferences of each individual (14), there is an imperative to understand *their* context from *their* perspective. Engaging caregivers is crucial too, because caregivers can also benefit from interventions directed toward both people in the caregiving dyad and actively engaged in interventions. They are often in the best position to facilitate behavior change in the person with dementia they provide care for (15).

Participation in health care may be enhanced with information provision, with older people desiring more information that is appropriate to their needs. Yet, health professionals perhaps undervalue this information or lack the skills necessary for effective communication with people who have dementia (16). Assumptions may also be made by health professionals that the person with dementia is unable to communicate their experiences and engage actively in the falls prevention planning dialog. This can result in well-intended but possibly paternalistic one-way attempts by health professionals and care staff to drive health-care-related decision-making (17). One model described in other areas of health designed to overcome these existing limitations is the use of a knowledge broker, a person who provides a link between research evidence and consumers and health-care professionals, building their capacity to make the evidence relevant for their context (18). This approach may enhance the adoption of falls prevention strategies especially if they are designed around the individual needs and preferences of person with dementia and their caregivers (19).

The aim of this paper is to identify the perceptions of people with dementia and their caregivers on falls prevention prior to an individualized intervention; and to contrast this with views post-intervention particularly related to the successes and challenges of adopting falls prevention strategies.

## Materials and Methods

**Figure 1:**
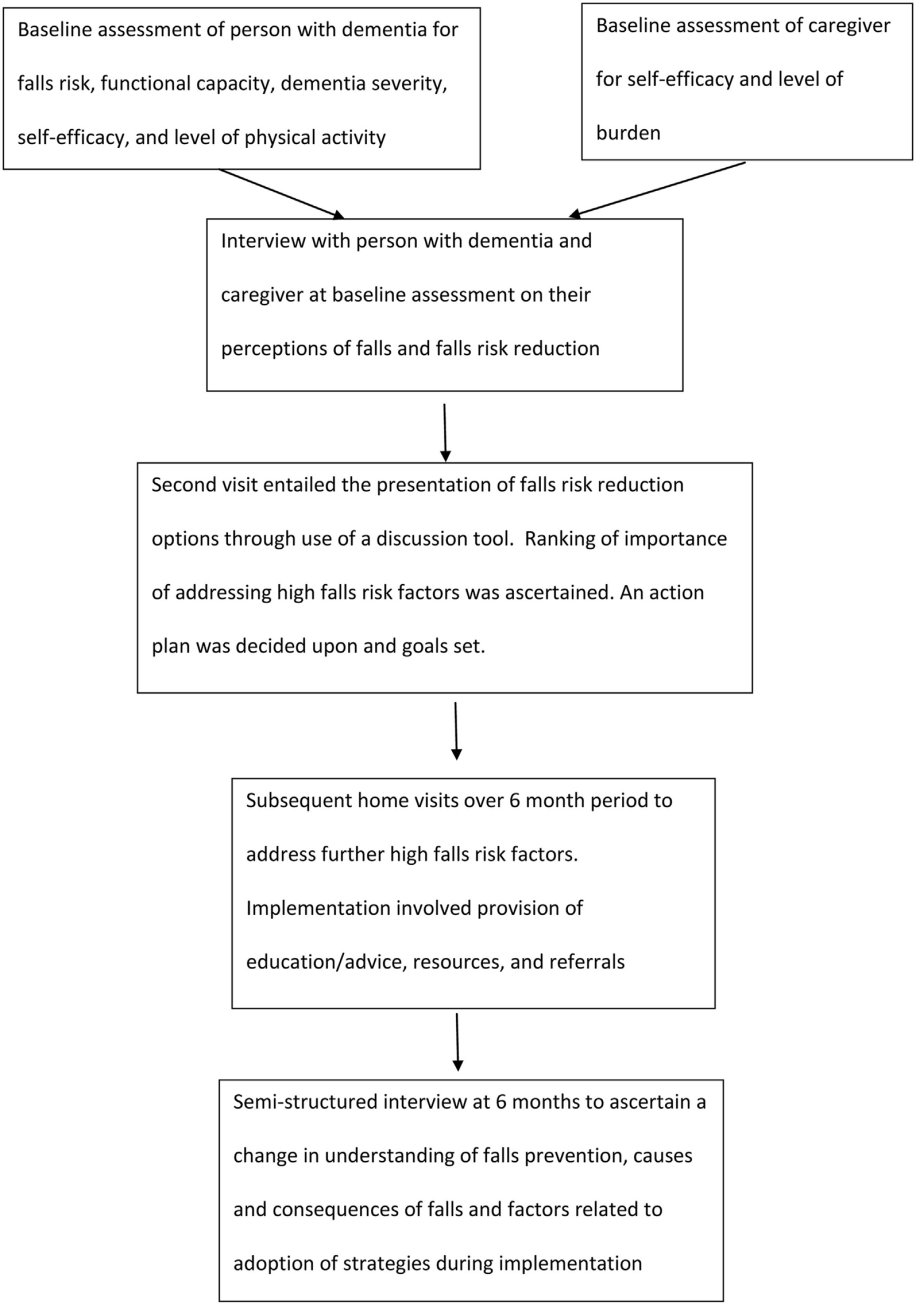
**Implementation of 6-month intervention**.

This paper reports the qualitative findings of a series of studies. The series of studies involved a 6-month intervention using assessment tools and a discussion tool to identify falls risk factors, rank risk factors according to agreed importance to change [between health professional (knowledge broker), the person with dementia, and their caregiver], provide options for falls prevention strategies, explain pros and cons of undertaking prioritized strategies, and support implementation. Further information regarding the full study methodology has been previously published (20), with Figure 1 highlighting the flow chart of intervention (results submitted for publication). Qualitative methodology, with a phenomenological approach, was used as it was appropriate to increase understanding of a complex and little researched area (21). Design, data collection, and analysis adhered to the Consolidated Criteria for Reporting Qualitative Studies (COREQ) (22).

### Participants

People with dementia and their caregivers were recruited through two community care agencies known to the study team; community events and support groups; snowballing techniques; personal and professional contacts; newspaper/newsletter advertisements; and known volunteer databases. As suggested by COREQ guidelines, potential participants were identified and approached by the agency, with details forwarded to the research team with participant consent. Inclusion criteria were (i) being over 18 years of age; (ii) having reasonable proficiency in English; (iii) having a diagnosis of dementia by a medical doctor; and (iv) a caregiver living with the person with dementia or visiting at least 2 days per week. A participant information sheet was provided and written consent obtained from both the person with dementia and the caregiver (or in the case of inability to consent, by the person responsible). Ethics approval was granted by La Trobe University Human Research Ethics Committee (HREC 12-017).

### Data Collection

A semi-structured interview was conducted at the baseline assessment (focused on understanding perceptions of and meaning attributed to, falls prevention) (see Box 1 for interview questions/prompts) and at 6 months (focused on whether there had been a change in knowledge/understanding of falls prevention and the factors related to adopting falls prevention strategies throughout the 6-month implementation with the knowledge broker) (see Box 2 for interview questions). Interviews were conducted in participants’ homes at a convenient time, the research process explained, and consent form signed (sent prior to the participants). There was the option of the caregiver and person with dementia being interviewed separately. The interviews were conducted by one of the research team (Claudia Meyer), an experienced, aged care physiotherapist who did not previously know the participants. Interviews were audio-taped with a digital recorder and field notes taken by the researcher. Data saturation was deemed to be reached when no further unique information was revealed. Findings from both interview occasions are presented sequentially, with the discussion highlighting change over time for participating dyads.

Box 1 **Interview questions for baseline assessment.**

- Can you tell me what you understand about falls? (use prompt of “falls are defined as unintentionally coming to the ground or some other lower level” if needed)
- Are falls important to you? Why? (more concrete version: are you worried about falling?)
- How does it make you feel when you fall? (more concrete version: how did it make you feel when you fell on the back step?)
- What can you do to decrease your risk of falls? (more concrete version: what can you do to stop yourself from falling?)
- What can others do? (more concrete version: what can “x” do to help you?)
- Have you been given any information about falls prevention in the past? (use prompt of “falls prevention information such as information about exercise for balance and strength, home modifications, or changing medications” if needed)
- Who gave you the information? (use prompt of “such as from your doctor, district nurse, physiotherapist, or occupational therapist” if needed)
- What did you think about the information provided?
  ○ Was the information helpful?
  ○ Were there any difficulties in using the information?
- (Carer possibly to answer the last three questions – more concrete example, when information is known, is: what did you think about the … information that … gave you?)
- What do you feel would be useful to you personally for falls prevention? (more concrete version: what would help you to stop falling?)

Box 2 **Interview questions for 6-month assessment.**

- Can you tell me what you understand about falls? (use prompt of “falls are defined as unintentionally coming to the ground or some other lower level” if needed)
- What can you do to decrease your risk of falls?
- What can others do?
- What motivated you to participate in the falls prevention program?
- What has worked well for you in preventing falls over the past 6 months?
- What have been the difficulties over the last 6 months of putting falls prevention into practice?

### Data Analysis

Data from people with dementia and their caregivers were combined for one overall thematic analysis, although data were kept separate by time period of baseline and 6 months. The audio-tapes were transcribed verbatim with transcripts reviewed several times (Claudia Meyer). Two independent reviewers (Claudia Meyer and Jean Tinney) used open coding initially, with interview questions as a guide, to identify recurring patterns within the text (23). Codes were applied to meaningful chunks of data and then grouped according to similarities and differences, contextualized with emerging phenomena (24). A third reviewer (Briony Dow) was included where there were discrepancies. Field notes were used to clarify interview responses and provide extra contextual information. Participant checking was not used given the nature of memory impairment for people with dementia.

## Results

### Participants

Thirty-nine people with dementia/caregiver dyads expressed initial interest in participating in the study, with a final sample size of 25 participant dyads at baseline. At 6 months, 24 caregivers and 16 people with dementia participated in interviews. Interview duration ranged from 20 to 45 min. Reasons for non-participation at baseline were person with dementia shortly to enter residential care (*n* = 5); caregiver too busy (*n* = 3); caregiver not interested (*n* = 3); caregiver did not feel the need (*n* = 1); and person with dementia did not have a caregiver (*n* = 1). Reasons for non-participation of people with dementia at 6 months were death (*n* = 3); moved into residential care (*n* = 3); loss of ability to communicate in English language (*n* = 1); and did not wish to participate (*n* = 1). Two dyads withdrew from the study due to death of the person with dementia. Reason for non-participation of one dyad was death of the caregiver. At baseline, 13 people with dementia were males (mean age of 80 years), 16 caregivers were females (mean age of 72.5 years). Dementia diagnosis was Alzheimer’s disease (*n* = 15); dementia with Lewy-bodies (*n* = 3); vascular dementia (*n* = 2); frontotemporal dementia (*n* = 2); and mixed dementia (*n* = 3). The median number of falls for people with dementia in the preceding 12 months was 1.0 (IQR 0, 2.5), with a large number (*n* = 16) reporting at least one fall within the 6-month intervention period, equating to 5.4 falls per person per 1000 days.

### Thematic Analysis

Five themes were identified from baseline interviews: *perceptions of falls; caregivers navigating the new and unpredictable; recognition of decline; health services – the need for an appropriate message; and negotiating a respectful relationship*. Similar themes, but with important differences noted, emerged from the 6-month interviews: *what we need to know; the right way* … *at the right time; more than just empty vessels to be filled; drawing on a variety of resources; and adapting to change*.

### Baseline Interview Findings

#### Perceptions of Falls

Participants perceived falls as unanticipated with a sense of nihilism and fatalism that falls just happen and nothing can be done about them.

for an unwanted reason you hit the ground (P10)^1^

not really a fall … overbalanced (P10)

They blamed the environment or themselves, with the caregiver and person with dementia at times at odds regarding the reasons for falls.

“rugs on floors” … “nothing slippery” (C13)

it was my fault that I didn’t go to the toilet earlier (P2)

“it is only half a fall if you are already on the ground … different to falling from a ladder” (P24) contrary to the caregiver’s view of “you can be kneeling in the garden and still fall … you are only perceiving it from the injury you might get” (C24)

Consequences ranged from being trivial, neutral, or catastrophic, but the overwhelming feeling attributed to falls was negative including for those with advanced dementia.

never hurt nothing … never broke nothing … so it’s OK (P10)

I don’t worry about it, but I pay attention (P7)

a major fall … it is the beginning of the end (P2)

a nasty feeling (P21)

made me feel useless (P10)

there are bad falls and there are badder falls (P18)

#### Caregivers Navigating the New and the Unpredictable

Falls prevention was challenging for caregivers of people with dementia, as they were often navigating unfamiliar territory, given the changes in ability and behavior of the person they cared for. As the same time, they were uncertain about the safest, yet most empowering, pathway for the person with dementia. Caregivers drew upon their prior knowledge, experience, and support received from others but at times felt it very overwhelming and at a loss as to where to begin.

I heard and listened to them … I could see the reason for it (C5)

advice from you as this is where we are at … we haven’t stopped to think about it (falls) (C17)

once you enter this area many people … it was for me just about information saturation … there was a time when it was bewildering (C23)

#### Recognition of Decline

Caregivers expressed growing concern for the person with dementia with recognition of physical and/or mental decline but were uncertain about addressing this decline from a falls prevention perspective and how to maintain independence and/or activity levels.

each thing that comes along I handle pretty well and I am interested in each thing … but I am taking each one as it comes because I can’t handle what is still coming down the road (C18)

the way she moves it … I don’t think it is right … but even if I move it back she will get up and move it (C5)

[trying] to get her to carry a stick, but she won’t (C5)

he can’t seem to understand what I want him to do (C25)

always been an exercise person … but now harder (C1)

#### Health Services – The Need for an Appropriate Message

Health services were at times considered to offer a bewildering array of service and support options, but most important was the need for information and support provided in an accessible, appropriate, and timely manner. For the person with dementia, the manner in which the message is delivered is important.

it all happening at once … perhaps it comes at a time when you are already very stressed about it and then trying to take all of this in just adds to the burden (C23)

all the information … we all have the knowledge in there, but to convey it at the acceptable level is the important thing … and at the appropriate time … I think that is the key of it all (C18)

taking a while to absorb everything … I am on a very steep learning curve (C19)

I thought at the time, but I just can’t recall … with most of the things she said I have completely forgotten … but she was quite good (P7)

#### Negotiating a Respectful Relationship

A negotiated respectful relationship, based on open communication and participation between people with dementia, their caregiver, and health professionals was considered desirable, with recognition of everyone’s unique contribution.

good to have the knowledge from someone who has the education to help you become educated about the situation (C5)

I shouldn’t say this, but we are not silly (C19)

we’ve had experience in life (C9)

learning as we go along (C17)

all part of the journey of being in it together (C10)

### Six-Month Interview Findings

These findings arose from interviews with people with dementia and their caregivers after the 6-month intervention, particularly related to adoption of falls prevention strategies (see Figure 1).

#### What We Need to Know

Following the 6-month intervention, falls prevention knowledge fell broadly into two categories: first, an understanding of falls risk and relevant risk reduction strategies and second, how the dementia process impacted falls prevention and its direct relevance for the person with dementia.

Both caregivers and people with dementia readily identified intrinsic and extrinsic falls risk factors.

He gets a bit giddy sometimes because of the medication and when he bends down to pick something up that can have an adverse effect (C1)

Flat heels, lace up shoes … I have got the nice T-bar ones … you go out and it doesn’t matter how dressed up you are, you just shove these shoes under the table … nobody is tottering around on high heels any more (P6)

Falls risk reduction strategies predominantly focused on improving balance, reducing environmental hazards, and medication use.

When you get up you have to wait a few seconds to get your balance and then take off’ … ’the most important thing for me, I think, is the exercise program (C5)

One of the biggest things is getting rid of these mats … he can go all the way around the house now without any steps (C1)

He (the GP) was very supportive in that I wanted to take her off the Simvastatin [a medication] and a few other things (C11)

Caregivers also identified falls risk and/or strategies that were particularly pertinent to the person with dementia, acknowledging the unique concerns facing the people they were caring for, without the evidence of nihilism and fatalism as described at baseline.

His depth perception is out of whack … he can’t see where to put his bum (when sitting down) (C1)

I really couldn’t change it too much because that was what Mum was most familiar with … and if I changed anything too dramatically she was quite upset (C11)

With the gymnasium being too difficult … you have suggested the exercises so that is really good … so the focus has changed and we do more simple things (C19)

#### The Right Way … at the Right Time

The sharing of falls prevention knowledge is more complex than purely the provision of information. The nature and interconnectedness of information and service provision, acknowledging individual needs and preferences, is vital to effective knowledge translation.

For the more proactive caregiver, all information was appreciated, which helped them to connect various concerns and prepare in advance.

Information, whether it is relevant at the time or not, is going to be of some use to us … I think the more information you have the better equipped you will be to deal with the issues … because [P1] symptoms are not just Parkinson’s, they are Parkinson’s and dementia … I have been reading that they go hand in hand … it is not like falls are the biggest problem he has got, but falls are part of the overall condition that he has got (C1)

They [community care health professionals] are actually preceding my queries … foreseeing problems before I am actually seeing most of them … more preventive work, which is very good because often within a month or two things come along (C18)

There were those who appreciated service provision within the home environment, yet others who preferred the social benefits of group settings.

I think that it is marvellous where you can stay in your own home and they come and check you out and tell you what to do (P20)

It’s been quite good (balance exercise class) … it’s quite amazing the combination of getting together with the other ladies as well (P7)

Information and/or service provision modification was made for those who found it too overwhelming, with adaptation crucial to prevent cessation of a falls risk reduction strategy.

The physio[therapist] who came, I don’t think she realised how advanced the dementia was and so I think some of the exercises were too difficult and when you checked them you agreed … so we have broken them down and we just do the 8 or 10 that suit him (C19)

You’ve just got to watch that he doesn’t get tired … when he gets tired his Alzheimer’s ramps up and he doesn’t know where he is (C4)

#### More Than Just Empty Vessels to Be Filled

People with dementia and their caregivers expressed the desire to be an integral part of managing falls, through a shared decision-making process with the knowledge broker. While grateful for information provided to them, particularly in the manner outlined in the right way at the right time, there was a definite preference for seeking recognition of *their* knowledge and how that knowledge fitted within the context of *their* lives.

having someone come to the house that is not fully aware of what the situation for (person’s name) is like … not being prepared for the possibility of a fall is an issue … they don’t tell the people who are coming to the home enough about what the situation is truly like (C1)

She won’t do the exercises when she feels tired or I think when she feels a bit fatigued, or when her heart is racy … she just wants to be left alone to rest, but otherwise she is happy (to do exercises) (C5)

Caregivers valued being “part of the team,” with their knowledge of the person with dementia critical to implementing falls prevention strategies.

A man of his own will … he won’t listen … at least to me he won’t listen, but to an outsider he will listen (C3)

He started off by feeding himself [in respite] but by half way through it he was being fed … I don’t say that they did anything wrong, but it just … whether it was my not being there that upset him (C27)

The person with dementia, too, understood falls prevention in their context.

Brain wants to go one way and you want to go the other way … but I still find if I talk to myself I do alright (P10)

He picked up that something was wrong with the tablets and there was an extra one in there … and the nurse said he was right, and she rang the dispensary and there was an extra tablet in there that shouldn’t have been there (C25)

#### Drawing on a Variety of Resources

Within the community health-care sector, there are a variety of resources to draw upon for both falls prevention and dementia care. Medical specialists, allied health professionals, community care managers, and direct care workers interconnected by a knowledge broker all provided a solid basis for falls prevention strategies to be implemented throughout this series of studies. The interconnectedness of health professionals and services offered a more holistic and integrated approach to care.

I have (found it useful) … certainly everything from the dietician, the memory clinic … yourself that you arranged those things from the dietician and (name) Community Health setting us up … because I didn’t know where to go … I didn’t have a clue what to do with any of the things (C16)

So far everything has worked out pretty well … and the bit of extra knowledge that I have now has taken the unknown away a bit so I won’t be surprised … and then I can always go to the person concerned for help … and that is nice to know (C17)

#### Adapting to Change

Caregivers regularly expressed the need to adapt to change: change in their knowledge and understanding of caring for the person with dementia with a focus on falls prevention, change in the presentation of dementia, and change in routines.

I have changed the way we are getting dressed … the way we do our showering (C1)

Trying to look at things from a different angle … well, that is probably what he tried to do when he fell between the bed and the wall because that would be the side that he would (normally) get out of … of course you don’t think of these things until you study it (C27)

At [name of group] now they have got a walker for you there so that you don’t have to take yours across … they make sure that … well, that he is able to … the toilet that is normally used as a store room, they make sure that it is clear so that [person’s name] can get in there (C10)

Like the weight loss … and the importance of his feet … they are just things that you take for granted and now that you have come and spoken with us about it, it is something that I have thought about a little more (C15)

These adaptations allowed for increased awareness and management of falls risk, with information empowering them to make changes.

more conscious to what you can do and what you can’t do now … you sit down and work it out another way (P10)

it makes a big difference when you are not worrying about different things (P22)

## Discussion

The uniqueness of this study was in capturing the voices of people with dementia and their caregivers prior to and following a 6-month intervention, specifically around the adoption of falls prevention strategies. Five themes emerged from the baseline interviews, highlighting the variable knowledge regarding falls risk factors and prevention strategies, the unpredictable and often challenging journey of seeking falls prevention advice, and the desire for a respectful health-care partnership. Five additional themes emerged from data collected after 6 months of an intervention, targeting individualized strategies for high falls risk factors with the assistance of a knowledge broker, but there were some important changes, perhaps suggesting that their perceptions had changed over time and may have been impacted by the intervention. At 6 months, caregivers and people with dementia were much clearer about “*what we need to know*” with firm views that the information regarding falls risk reduction needed to be in “*the right way* … *at the right time*.” Rather than caregivers and people with dementia being only recipients of knowledge, they felt they were “*more than just empty vessels to be filled*” drawing on a “*wealth of resources*” within their circle of influence to be able to positively “*adapt to change*.” These themes have been further synthesized in order to provide three key messages for health professionals to take note of to increase uptake of falls prevention strategies among people with dementia. These are respecting the person with dementia and their caregiver; meaningful engagement and shared decision-making; and effective and timely communication with a trusted source. These insights provide a framework for community care health professionals to understand that people with dementia and their caregivers can, and wish to, contribute to implementing falls risk reduction strategies, particularly with knowledge requested “*the right way at the right time*.”

### Respecting the Individual

Themes of “*what do we need to know*,” “*more than empty vessels to be filled*,” *and* “*adapting to change*” all contributed to the message of respecting the individual. Respecting the individual person with dementia and their caregiver, respecting the context of their lives in which falls prevention strategies are to be implemented, are crucial for, and consistent with, principles of person-centered care. Person-centered care involves generating shared values (25), shared power, and responsibility in decision-making (26). Drawing on the work of Kitwood (27) in the context of falls prevention, understanding personhood, that is, recognition and respect of the person, will allow both caregiver and health professionals to consider prior experiences and preferences, adapt to the changing needs of the person with dementia from which a prevention program can be formulated. At the commencement of the study, caregivers spoke of how overwhelming it can be to navigate the constantly changing needs and capacities of the person they care for especially in relation to falls prevention. They also spoke of how they are often given information when they are most stressed and therefore least able to make use of it. At 6 months, under the theme of “*what do we need to know*,” caregivers acknowledged the unique concerns facing the person they were caring for, a constant moving landscape. The theme of “*more than empty vessels to be filled*” highlighted the desire for a negotiated and respectful relationship between the caregiving dyad and health professionals, with more proactive caregivers appreciating being included in the knowledge sharing process. Caregivers have specific knowledge regarding the circumstances of previous falls, what works, and doesn’t work in the unique context of their lives. This was more evident at 6 months through “*adapting to change*” according to what was required. A sense of agency emerged particularly for the more proactive caregivers. The findings of this study support the work of McIntyre and Reynolds (28), whereby caregivers described learning as they went along to navigate the impact of falls and maintain the *status quo* in an ever-changing environment. Caregivers have a unique perspective on developing a falls prevention plan that may be crucial in the successful adoption of falls prevention strategies.

### Meaningful Engagement and Shared Decision-making

The themes of “*the right way* … *at the right time*” and a “*variety of resources*” expressed by interview participants at the 6-month time point showed how important meaningful engagement in falls prevention strategies and having support from health professionals in decision-making was to these participants. This was reinforced through the theme of “*more than empty vessels to be filled*,” which illustrated the desire for people with dementia and their caregivers to be recognized for their knowledge and how that knowledge impacts on how they take up information about managing falls risk. Engagement may be enhanced by strengthening the older person’s involvement in health care and understanding their perspective (29). Interestingly, the nihilism and fatalism toward falls and falls prevention mentioned in the baseline interviews was not expressed at 6 months, suggesting a greater sense of empowerment at 6 months through connections with health professionals (including the knowledge broker) and other resources.

Perception and management of falls risk by health professionals tends to follow a risk discourse, with causes of falls often attributed to the individual, and the assumption that the person who has fallen is vulnerable, needy, and responsible for their own risk (30). Health professionals merely stating the falls risk factors and/or action to be undertaken, with little understanding of the person’s context, may inadvertently reduce the level of engagement by people with dementia and their caregivers with falls prevention strategies. This study involved shared decision-making with a knowledge broker to address falls risk factors, with participants expressing the value of interconnectedness of services and recognition of their own skills and capabilities. The use of a discussion tool, as used in this study, adds weight to the perspectives of people with dementia and their caregivers, allowing for frank discussion regarding *their* needs and *their* preferences in the context of *their* lives.

Caregivers play a pivotal role in interventions such as in this study. Caregivers provide encouragement to undertake risk reduction strategies (29), particularly important for a person with dementia with variable memory capacity. They also physically assist with exercise programs (10) and play a role in negotiating hazard reduction and risk-taking behaviors (31). The caregiver’s role in falls prevention is increasingly important as the dementia process continues and, while this role is often reliant on a caregiver’s personal characteristics, it may actually relate more to active engagement of the caregiver and method of intervention delivery (15). Research conducted by Gitlin and Rose (32) showed caregiver readiness to change behavior for an intervention targeted to the person with dementia was related to their willingness to engage with, and perceive the positive benefits of, the intervention, more so than any personal characteristics. Engaging meaningfully, through education and skill-building, can significantly reduce the behavioral and psychological symptoms of dementia and the caregiver’s response to these symptoms (33). Caregivers in this study emphasized the value of being part of the health-care team, with the right information provided at the right time so that they could make an informed decision with the context of *their* lives. This potentially impacts the design of falls prevention strategies and approaches to optimize implementation for this population. Falls prevention strategies are numerous and, at times, potentially overwhelming. A knowledge broker, the link between the research evidence and application of the evidence into practice for people with dementia and their caregivers, with the use of a discussion tool, can effectively engage people to prioritize falls risk factors and prevention strategies of importance to them.

### Effective Communication

Effective and timely communication was clearly expressed as a need by participants in this study, including style and delivery of the communication, to ensure understanding while simultaneously avoiding information overload. Prior to the intervention, participants expressed their appreciation for information but, critically, expressed the need for knowledge to be “conveyed at an acceptable level, at an appropriate time.” Encouraging active participation and decision-making in the translation of falls prevention knowledge relies on effective communication (12). Increasing knowledge begins with information delivered in a timely and appropriate manner (29) to ensure personal relevance and, importantly, not being patronizing or anxiety provoking (29). Falls prevention advice has the potential to imply personal responsibility for falls risk (30), that a person could be doing more to avoid falling (29). At 6 months, the nature and interconnectedness of information, which acknowledged individual needs and preferences, became evident through the theme of “*the right way* … *at the right time*.” Health professionals were important in identifying issues and providing individualized strategies leading to greater confidence in managing falls as evidenced through “*adapting to change*.”

Existing systems of information provision and communication between community care health professionals/community care staff and people with dementia and their caregiver were considered somewhat limited by the participants in this study. A knowledge broker may assist with the social nature of bridging the divide between research evidence and effective action (34) and may enhance participation of the person with dementia and their caregiver in the adoption of falls prevention strategies. In this study, intervention delivery occurred through a knowledge broker and was focused on strong partnerships with, and authentic involvement of the person with dementia and caregivers (35). The knowledge broker within this series of studies provided a source of support, reassurance, and guidance for the caregiver and, at times, the person with dementia as they navigated the unpredictable journey associated with increased falls risk and the ongoing dementia process. The challenges of caring for a person with dementia, of which falls are a part of the dynamic of health-care needs and conditions, may result in increased burden, decreased quality of life, depression, and even increased risk of mortality (36, 37). An intervention interconnected with a knowledge broker is a potential mechanism for the provision of timely and appropriate information and choice in falls prevention strategies. Strategies can be readily linked with the changing dynamic of the dementia process with the knowledge broker acting as a channel through which to connect health-care resources and information “*at the right time*,” thus sustaining the dyad in their role as long as is feasible.

A study limitation is whether the results can be generalized beyond the community care population studied, but with data saturation reached, unique insights for this population were revealed. The role of the researcher should also be acknowledged as a limitation, with the researcher collecting, collating and interpreting the data through a particular lens.

The inclusion of a knowledge broker was a key component of this study, with the potential for this role to be incorporated within existing community care structures to ensure the efficient and effective translation of falls prevention knowledge. Key recommendations to emerge from this study regarding the knowledge broker role are that the knowledge broker requires the following:

- A solid understanding of falls risk factors and prevention strategies, including the variety of resources available to people with dementia and their caregiver. For example, the Dementia Enabling Environments website allows for consideration of environmental hazards from the perspective of the person with dementia.
- An ability to respectfully and meaningfully engage the person with dementia and their caregiver in a health-care partnership, acknowledging individual needs and preferences, prior knowledge, and experience. The use of a discussion tool as proposed by this study allows for this to occur.
- An ability to convey the falls prevention message in an appropriate and timely manner, being vigilant for stress and overload of information. For example, to acknowledge the timing of a diagnosis of dementia and being mindful of the plethora of information given at the time of the diagnosis. As per the protocol for the full study, working through risk factors with people with dementia and their caregivers when they are ready to address them may be of benefit.

## Conclusion

This study has expressly sought the unique perspectives of people with dementia and their caregivers. According to study participants, falls risk reduction messages are best tailored to individual needs and preferences, and prior knowledge and experience. Information is best delivered in a timely and appropriate manner. Identification of whether a person with dementia and/or their caregiver are unaware of or underestimating falls risk; unable or unwilling to yet commit to change; or are ready for an action-oriented strategy may impact the success of addressing a falls risk factor. Inclusion of all parties in the decision-making process, with open communication and respect for each other, will enhance the delivery and receipt of the falls risk reduction message.

## Author Contributions

CM, BD, KH, JT, and SH have all substantially contributed to the conception or design of the work and the acquisition, analysis, and interpretation of data for the work; have all assisted in drafting the work or revising it critically for important intellectual content; have given final approval of the version to be published; and agree to be accountable for all aspects of the work in ensuring that questions related to the accuracy or integrity of any part of the work are appropriately investigated and resolved.

## Conflict of Interest Statement

The authors declare that the research was conducted in the absence of any commercial or financial relationships that could be construed as a potential conflict of interest.

## Footnotes

^1^“P” is used to denote a quote by the person with dementia, and “C” is used to denote a quote by the caregiver.
